# Neutrophils at the Crossroads of Inflammatory Bowel Disease and Atherosclerosis: A State-of-the-Art Review

**DOI:** 10.3390/cells14100738

**Published:** 2025-05-18

**Authors:** Vadim Genkel, Yana Zaripova, Alla Kuznetsova, Alena Sluchanko, Anna Minasova, Maria Zotova, Anna Saenko, Albina Savochkina, Anastasiya Dolgushina

**Affiliations:** 1 Department of Internal Medicine, South-Ural State Medical University, Chelyabinsk 454141, Russia; yanakud123@mail.ru; 2 Department of Hospital Therapy, South-Ural State Medical University, Chelyabinsk 454141, Russia; kuzja321@mail.ru (A.K.); pauttova@yandex.ru (A.S.); anna-selyanina@mail.ru (A.S.); dolgushinaai@yandex.ru (A.D.); 3 Research Institution of Immunology, South-Ural State Medical University, Chelyabinsk 454141, Russia; pandora_anna@mail.ru (A.M.); zotova.chel@mail.ru (M.Z.); alina7423@mail.ru (A.S.)

**Keywords:** inflammatory bowel disease, atherosclerosis, neutrophils, cardiovascular disease, inflammation, ulcerative colitis, Crohn’s disease

## Abstract

Inflammatory bowel disease (IBD) is a growing global problem, particularly in regions with low sociodemographic indices and growing populations. IBD incidence is increasing among children and adolescents, leading to a growing economic burden. The prevalence of atherosclerotic cardiovascular diseases among patients with IBD is also higher than in the general population. While mortality rates have decreased, cardiovascular disease (CVD) remains a significant contributor to mortality and disability in IBD patients. According to the current understanding, neutrophils play an important role in both the atherogenesis and pathogenesis of IBD. This review addresses the state of the art of neutrophil involvement in the development of atherosclerosis and IBD. In the present review, we summarize the currently available evidence regarding neutrophils as a possible key driver of extraintestinal manifestations of IBD and cardiovascular complications. We provide a discussion on the potential role of neutrophil-derived markers in the development of new approaches for the precise diagnosis of atherosclerosis in patients with IBD, as well as new therapeutic targets.

## 1. Inflammatory Bowel Disease (IBD) and Atherosclerosis: “Real-World” Data and Clinical Trial Results

Inflammatory bowel disease (IBD) is a global medical and social problem, and its prevalence is likely only to increase in the coming decades. This issue will be especially pertinent for regions with low sociodemographic index values and growing populations, such as South Asia, Eastern Europe, Latin America, and Oceania, where there is a continued increase in the incidence of IBD [1,2,3]. Another important epidemiological trend is the increasing incidence of IBD among children and adolescents living in these regions (as well as in countries with high sociodemographic indices), which, together with population growth, increasing life expectancy, and decreasing IBD-related mortality, will lead to a growing economic burden from IBD in developing countries [4,5,6].

The results from nationwide studies indicate a significant decrease in mortality in patients with ulcerative colitis (UC) and Crohn’s disease (CD) over the past 30 years [7,8]. However, at least in certain categories of patients with IBD, there is still an increase in the relative risk (RR) of all-cause mortality compared with the general population: patients with IBD onset before the age of 17; and male patients with CD onset after 40 years of age, who have colonic disease and a penetrating phenotype [9,10]. Cardiovascular diseases (CVDs) make an important contribution to the structure of mortality and disability in patients with IBD; however, according to some data, cardiovascular mortality in patients with UC and CD significantly decreased and stabilized from 1999 to 2019 [10,11,12,13]. According to the IBSEN study (Inflammatory Bowel Disease in South-Eastern Norway), which included 756 IBD patients, the RR of cardiovascular death over 28 years of follow-up was significantly higher in both UC patients (HR 1.51 (95% CI 1.10–2.08)) and CD patients (HR 2.04 (95% CI 1.11–3.77)) compared to the controls [10]. According to a meta-analysis of 11 clinical studies, patients with IBD have an increased risk of coronary artery disease (CAD) (RR 1.17 (95% CI 1.07–1.27)), myocardial infarction (RR 1.12 (95% CI 1.05–1.21)), and cerebrovascular disease (RR 1.25 (95% CI 1.08–1.44)) [14,15]. The prevalence of atherosclerotic cardiovascular disease (ASCVD) in patients with IBD is also higher than in the general population. According to an analysis of US National Health Interview Survey responses, containing data on 66,610 participants, among whom IBD was established in 951 patients, the age-adjusted prevalence of ASCVD was 12.0% compared with 6.9% in the general population (*p* < 0.001) [16]. At the same time, VPCs were associated with the presence of ACCP (OR 1.58 (95% CI 1.17–2.13)), after adjustment for demographic and traditional cardiovascular risk factors, mainly due to the category of patients younger than 64 years of age and female patients. 

These data are also supported by the results of an analysis of the VITAL (Veterans With Premature Atherosclerosis) registry, which included more than 147,000 participants, among whom 9485 patients were diagnosed with premature or extremely premature ACSS (ACSS aged ≤55 years and ≤40 years, respectively) [17]. Compared with the control group, patients with HCC had a significantly increased OR of extremely premature ACSS (OR 1.61 (95% CI 1.34–1.94)), which was more pronounced among patients with UC (OR 1.53 (95% CI 1.12–2.08)) compared with patients with CD (OR 1.35 (95% CI 1.00–1.82)). An increased risk of coronary heart disease (CHD), including premature CHD, has also been demonstrated by the results of the United Kingdom Biobank analysis and a multicenter study in China [18,19]. Thus, the results of the UK Biobank analysis showed a significant 1.18-fold (95% CI 1.06–1.32) increase in the HR of CHD over 12.4 years of follow-up and a 1.32-fold (95% CI 1.03–1.69) increase in premature CHD over 8 years of follow-up.

It should be briefly noted that data on the prevalence of traditional cardiovascular risk factors and on the magnitude of their impact on the development of ASCVD have been inconsistent to date [16,20]. However, more than half of IBD patients are likely to have an unfavorable overall cardiovascular risk factor profile [21]. Specifically, factors that are difficult to assess correctly in population-based studies evaluating electronic medical record data, such as physical inactivity, sleep disorders, and psychosocial distress, are likely to be important contributors to cardiovascular risk in patients with IBD [22,23,24]. Collectively, these factors are closely associated with obesity, observed in up to 40% of IBD patients, which both influences the risk of atherosclerosis and the course of IBD [25,26]. There is growing intriguing evidence that the melanocortin system may play a critical role not only in the development of obesity and hypertension, but also has a potential immediate impact on the course of IBD and atherosclerosis [27,28,29]. Another important factor that can modify cardiovascular risk in patients with IBD is the administration of disease-specific therapies. According to currently available data, taking into account conflicting results from several studies, thiopurines and TNF inhibitors may favorably affect cardiovascular risk, corticosteroids, tacrolimus, and IL-12/23 inhibitors, and JAK inhibitors may increase the risk of adverse cardiovascular events, while aminosalicylates have no significant effect on cardiovascular risk [30].

The data on the prevalence and characteristics of subclinical atherosclerosis in patients with IBD are limited. In a study by A. Hernandez-Camba et al., which included 186 IBD patients and 175 control group patients, a higher frequency of carotid atherosclerotic plaque was found in the subgroup of low CVR patients with IBD compared with the control group (21% vs. 11% (*p* = 0.034)) [31]. In contrast, a study by R. Naami et al. showed that the frequency of subclinical atherosclerosis of the coronary arteries, assessed using the coronary artery calcium score, did not significantly differ between patients with IBD and the control group [32]. This result is consistent with the results of another study, in which the frequency of abdominal aortic calcification did not significantly differ between patients with IBD and the control group (35.7% vs. 30.6%, *p* = 0.448) [33]. At the same time, subgroup analysis showed that IBD patients with established CVD, as well as UC patients, had a higher frequency of severe abdominal aortic calcification compared with IBD patients without CVD and CD patients, respectively (6.7% vs. 25.7%, *p* = 0.001 and 56.7% vs. 26.5%, *p* = 0.01).

Both the results of large epidemiological studies and small clinical studies assessing subclinical atherosclerosis have identified two critically important issues in assessing the risk of ASCVD in patients with IBD: (1) the limited diagnostic and prognostic value of standard approaches that demonstrate greater efficacy in the general population; and (2) the incorporation of clinical, laboratory, and endoscopic markers of IBD activity may increase the effectiveness of standard approaches to ASCVD risk assessment [34,35]. According to modern concepts, neutrophils play an important role in both the atherogenesis and pathogenesis of IBD. Clinical trial results also indicate that neutrophil counts are associated with the risk of ASCVD in patients with IBD [19]. Studying the role of neutrophils as key participants in the progression of atherosclerosis and intestinal lesions in patients with IBD may contribute to the identification of new therapeutic targets, as well as the discovery of novel diagnostic and prognostic markers.

## 2. The Role of Neutrophils in Atherogenesis: From Associations to Causality

Neutrophils represent the most numerous leukocyte population, but the role of neutrophils in atherogenesis and the development of related complications have only been actively studied over the past few years [36]. This was largely facilitated by new discoveries in neutrophil biology that allowed for a revision of traditional views regarding their lifespan and the homogeneity of the population composition [37]. Single-cell RNA sequencing studies have established the plasticity of neutrophils at the level of chromatin, transcriptome, and proteome [38]. These data allowed for the identification of mechanisms that provide the phenotypic and functional heterogeneity of neutrophils, which, in turn, contributed to progress in the study of neutrophil heterogeneity in various diseases, including atherosclerosis [39,40].

Until recently, the results of clinical studies, including large epidemiological studies, have indicated associations between neutrophil counts and the risk of ASCVD, which could not confirm or refute causal relationships between neutrophil counts and atherosclerosis. Thus, according to the CALIBER (cardiovascular disease research using linked bespoke studies and electronic health records) study, which included 775,231 participants, an increase in the neutrophil count within the reference range was associated with an increase in the relative risk of developing heart failure (RR 2.04; 95% CI 1.82–2.29), atherosclerotic peripheral arterial disease (RR 1.95; 95% CI 1.72–2.21), an abdominal aortic aneurysm (RR 1.72; 95% CI 1.34–2.21), non-fatal myocardial infarction (RR 1.58; 95% CI 1.42–1.76), and coronary death (RR 1.78; 95% CI 1.51–2.10) [41]. According to an analysis of the UK Biobank, which included 478,259 participants, the risk of cardiovascular death among patients from the highest decile of the neutrophil count was significantly higher in both men (HR 1.59; 95% CI 1.22–2.08) and women (HR 2.15; 95% CI 1.38–3.35), which was also true for non-fatal CVD (HR 1.28 in men, 95% CI 1.16–1.42; HR 1.21 in women, 95% CI 1.06–1.38) [42]. However, the first convincing evidence of the causal role of neutrophils in atherogenesis was obtained only recently, in 2023. The results of a Mendelian randomization study were published, demonstrating that a genetically determined increase in the number of circulating neutrophils by one standard deviation is associated with an increase in the relative risk of CAD (OR 1.15; 95% CI 1.08; 1.21), myocardial infarction (OR 1.22; 95% CI 1.12–1.34), and atherosclerotic peripheral arterial disease (OR 1.19; 95% CI 1.04–1.36) [43,44].

Moreover, other articles describe various mechanisms of neutrophil involvement at all stages of atherosclerosis development: the release of proteolytic enzymes (cathepsins, matrix metalloproteinases, gelatinases, elastase), the release of reactive oxygen species, the secretion of pro-inflammatory cytokines (TNFα, IL-1β, IL-1ra, IL-6, CXCL1, CXCL8, CXCL10, CCL2, CCL3, CCL4, CCL23, G-CSF, VEGF) and, alarmingly, the elevation of S100 calcium-binding proteins, the formation of neutrophil extracellular traps (NETs), and the recruitment of other immunocompetent cells into the vascular wall and cooperation of other cells with them [36,45,46,47,48].

Thus, there are currently several levels of evidence (mechanistic, epidemiological, and genetic) that neutrophils are an important causal factor in the development and progression of atherosclerosis.

## 3. The Role of Neutrophils in the Pathogenesis of IBD

The gastrointestinal tract functions in a state of chronic low-grade inflammation associated with the constant processing of antigens entering the lumen and the priming of the mucosal immune system to eliminate antigens that overcome the epithelial barrier [49]. Neutrophils are key players in the innate immunity of the gastrointestinal tract, and neutrophil infiltration of the intestinal mucosa is a characteristic feature of active IBD [50]. The main biomarker of IBD activity in clinical practice is neutrophil granule proteins, and the appearance of anti-granulocyte–macrophage colony-stimulating factor autoantibodies (aGMAbs), which are important for maintaining neutrophil homeostasis, preceding the development of CD in subsequent years [51]. In addition, neutrophils play an important role in the interaction with representatives of the intestinal microbiome, being at the forefront of the host–microorganism interface [50]. These findings indicate an important role of neutrophils in the pathogenesis of IBD; however, their role is currently known to be significantly less important than, for example, the role of adaptive immunity and T cells in the development of CD and UC [52].

According to current concepts, the roles of neutrophils in CD and UC differ significantly and can be described extremely simplistically as “dysfunction” and “hyperactivation”, respectively [50]. Thus, neutrophils in CD exhibit decreased oxidative and phagocytic activity, leading to impaired bacterial clearance and the activation of adaptive mucosal immunity [53]. In a cohort of pediatric patients with CD, it was shown that neutrophils from patients with a stricturing phenotype of the disease were characterized by decreased GM-CSF signaling, which, in turn, was associated with specific disorders in the regulation of cytokine production, repair, survival, and cell proliferation [54]. In contrast, in UC, unconstrained activation of neutrophils occurs, whose migration into the intestinal mucosa is associated with the development of chronic neutrophilic inflammation [55]. The assessment of neutrophil infiltration to evaluate the severity of UC enables the course of the disease and the effectiveness of therapy to be predicted to the greatest extent compared to other histological characteristics [56,57]. The severity of neutrophil infiltration into the intestinal mucosa, as well as the content of neutrophil-associated markers in it (S100A12, PROK2, FCGR3B, GPR109B), can serve as a valuable predictor of the development of colitis-associated colorectal cancer, which also indicates an important role of neutrophils in the course of UC [58].

However, despite the known differences in the immunopathogenesis of UC and CD, as well as the obviously different roles of neutrophils in their development and progression, the assessment of the neutrophil content in the mucosa, as a component of various assessment systems, as well as the assessment of the content of the neutrophil-associated biomarker, calprotectin, in feces, are common components of monitoring the course of both UC and CD. Among the promising serum markers of IBD, several neutrophil-associated markers also demonstrate diagnostic efficacy in both CD and UC. According to a review of 34 studies, involving 1850 patients with CD and 1122 with UC, several neutrophil-associated serum markers (LL-37, nCD64, CPa9-HNE, TREM1, eNAMPT) may be valuable in both UC and CD [59]. Important data are also provided by studies describing the transcriptomic landscape of peripheral blood in patients with UC and CD [60]. The study found that the peripheral blood transcriptome did not differ significantly between the UC and CD groups. At the same time, in IBD patients compared with the controls (both in the group of treatment-naive patients and in the group receiving therapy), there was a deregulation of mRNAs and microRNAs involved in the innate immune response and neutrophil activation (especially enriched in neutrophil activation-related pathways). In a study by V. van Unen et al., which aimed to identify a disease-associated network of intestinal immune cells, no significant differences were observed between UC and CD [61]. In both cases, a network of cells was found, including HLA-DR^+^CD38^+^ EM CD4^+^ T cells, T regulatory-like cells, PD1^+^ EM CD8^+^ T cells, neutrophils, CD27^+^ TCRγδ cells, and NK cells.

## 4. Neutrophils as Key Drivers of Atherosclerosis in Patients with IBD

The important role of neutrophils in the pathogenesis of chronic inflammatory diseases, including autoimmune diseases, as well as their key role in maintaining intestinal inflammation in both UC and CD, makes it possible to consider neutrophils as a possible key driver of extraintestinal manifestations of IBD and cardiovascular complications [62,63,64,65]. For example, in both IBD and osteoporosis, there is an upregulation of common NET-related genes, namely *HDAC6*, *IL-8*, *SRC*, *PPIF*, *PLCG2*, *PIK3CD*, *MAP2K1*, and *AKT1* [66]. The use of three of them (*HDAC6*, *IL-8*, *PPIF*) as a diagnostic tool for IBD, combined with osteoporosis, demonstrated high diagnostic efficacy (AUC 0.80). IBD and ankylosing spondylitis are characterized, among other things, by the upregulation of common differentially expressed genes associated with neutrophil activation (*SBNO2*, *DYSF*, *SRPK1*, *ACSL1*, *BCL6*) [67]. Among the skin diseases associated with IBD, pyoderma gangrenosum is probably associated with hyperactivation of IL-1β-primed neutrophils [68,69]. In experimental studies of acute ulcerative colitis induced by DSS administration in App^NL-G-F^ mice (a model of Alzheimer’s disease), an increase in the accumulation of neutrophils in the brain after the development of colitis was found [70]. At the same time, neutrophils were located in places of β-amyloid aggregation, and the suppression of their accumulation was achieved using an MMP-9 inhibitor, which also indicates the role of neutrophils in the progression of neurodegenerative disorders in patients with IBD.

As indicated above, in Section 2, genetically determined increases in the number of circulating neutrophils are associated with increased risks of developing ASCVD [43]. However, it is equally significant that an increase in leukocyte counts during life is also associated with an increased risk of adverse cardiovascular events. According to the Dongfeng–Tongji cohort study, which included 11,594 patients, an increase in the number of circulating leukocytes and neutrophils after 5 years of follow-up was independently associated with the development of adverse cardiovascular events [71]. Patients with an increase in leukocyte count, by more than 0.44 × 10^9^/L, had an increased risk of all types of cardiovascular events (HR 1.14 (95% CI 1.04–1.24)), CAD (HR 1.11 (95% CI 1.01–1.22)), and stroke (HR 1.26 (95% CI 1.03–1.55)). Comparable results were obtained in the Kailuan study, which included 61,666 patients, with a follow-up period of 6.65 ± 0.83 years [72]. Depending on the trajectory of the changes in their total leukocyte count, patients were divided into five categories: low–stable, moderate–stable, elevated–stable, moderate–increasing, and elevated–decreasing. Patients with a moderate–increasing pattern compared with patients with a low–stable pattern had an increased risk of developing CVD (HR 1.36; 95% CI 1.24–1.50) and myocardial infarction (HR 1.91; 95% CI 1.46–2.51), whereas patients with an elevated–stable pattern had an increased risk of all-cause mortality (HR 1.77; 95% CI 1.52–2.06). Both in experimental models of colitis and clinical studies, an increase in the number of circulating neutrophils is recorded at disease onset and during IBD flare, as well as in the preceding period [70,73,74,75,76]. It is hypothesized that a leading role in increasing the number of circulating neutrophils in IBD is played by the activation of the “IL-23–IL-17A” axis, which triggers the transcription of G-CSF in the bone marrow, followed by an increase in the production of CXCL1 and CXCL2, which lead to the release of neutrophils from the bone marrow [76]. In addition, pathways that trigger extramedullary myelopoiesis in patients with IBD have been described, which also contributes to an increase in the pool of circulating neutrophils [77,78].

Thus, from a mechanistic point of view, prolonged episodes of increased numbers of functionally activated neutrophils can serve as drivers of atherosclerosis progression [79,80]. From this perspective, the results from a study by Y. Ostendorf et al., in which the authors studied factors associated with the development of atherosclerosis in Apolipoprotein E-deficient mice with DSS-induced colitis, are important [81]. A synchronous increase in the circulating neutrophils and G-CSF concentration was observed after the second and third DSS cycles, as well as in the number of hematopoietic stem cells and myeloid progenitor cells. The circulating neutrophils demonstrated the upregulation of pro-inflammatory genes and a more adhesive phenotype, as determined by the expression of the Glg1 (Golgi Glycoprotein 1) and Selplg (Selectin P Ligand) mRNA. The use of anti-Ly6G, which contributes to a decrease in circulating neutrophils, led to a decrease in the burden of aortic atherosclerosis in mice, which also confirms the role of neutrophils as drivers of atherosclerosis.

**Figure 1:**
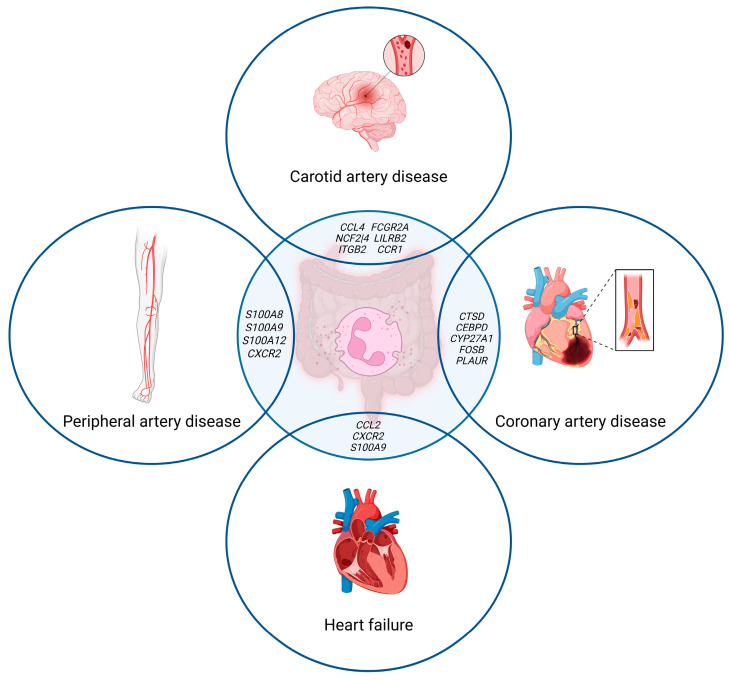
Shared neutrophil-associated genes involved in IBD and ASCVD [82,83,84,85,86]. Created in BioRender; Genkel, V. (2025); https://BioRender.com/zilb90s (accessed on 11 May 2025).

Important information about the role of neutrophils in atherogenesis in patients with IBD is provided by studies using bioinformatics analysis and machine learning to search for common hub genes (see Figure 1).

Thus, Z. Yao et al. identified common hub genes for CD and atherosclerotic disease of the lower extremities [82]. Among the 54 identified common differentially expressed genes, most were mainly related to pathways associated with neutrophil chemotaxis, neutrophil migration, and granulocyte chemotaxis. Based on the results from selecting hub genes among 15 potential candidate genes, four hub genes were selected that were significantly upregulated in both CD and peripheral arterial disease: *S100A8*, *S100A9*, *S100A12*, and *CXCR2*. The pathways associated with neutrophil activation, chemotaxis, and migration were significantly correlated with the high expression of *S100A8*, *S100A9*, *S100A12*, and *CXCR2* in CD and peripheral arterial disease. In the work by X. Tang et al., the hub genes for IBD and coronary heart disease were *CTSD (Cathepsin D)*, *CEBPD (CCAAT–Enhancer-Binding Protein Delta)*, and *CYP27A1 (Sterol 27-hydroxylase)* [83]. Correlation analysis between the genes and immune cells showed that these genes directly correlated with the neutrophil count and negatively correlated with CD4^+^ T cells. In the study by X. Luo et al., the hub genes for IBD and heart failure were *CCL2*, *CXCR2*, and *S100A9* [84]. According to the correlation analysis, *CCL2*, *CXCR2*, and *S100A9* were directly correlated with neutrophils, including activated neutrophils. Thus, the present results again indicate the critical role of pathways associated with neutrophil activation, migration, and chemotaxis, in the development and progression of atherosclerosis in patients with IBD.

## 5. Clinical Implications

**Figure 2:**
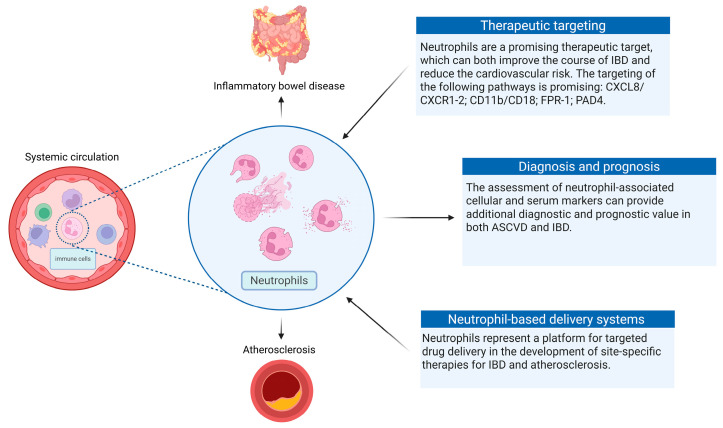
Clinical implications of recognizing neutrophils as key drivers of atherosclerosis in IBD. Created in BioRender; Genkel, V. (2025); https://BioRender.com/g6n6ohq (accessed on 11 May 2025).

The recognition of neutrophils as a key driver of atherosclerosis progression and IBD in patients with UC and CD has several important clinical implications (see Figure 2).

First, the assessment of neutrophil-associated cellular and serum markers, as well as markers representing pathways associated with neutrophil activation, migration, and chemotaxis, can provide additional diagnostic and prognostic value and contribute to the personalization of CVR assessment and monitoring in patients with IBD. Neutrophil-associated markers demonstrate diagnostic and prognostic efficacy in both ASCVD and IBD, and it can be expected that their use in IBD patients to assess CVR will be the most effective option [59,87,88]. In addition, this group of markers can be used to predict the response to IBD therapy [89,90,91].

Second, neutrophils represent a promising therapeutic target, which can both improve the course of IBD and reduce the risk of adverse cardiovascular events. A study of histomorphological features associated with a patient’s non-response to anti-TNF therapy and corticosteroids established the existence of high levels of neutrophil infiltration and fibroblast activation, which probably play a leading role in TNF-independent IL-1-mediated neutrophil chemotaxis [92]. Targeting IL-1 appears to be a promising approach in this category of patients, which has demonstrated efficacy in preventing atherothrombotic events in patients with atherosclerotic CVD [93,94]. Another potential therapeutic target is formylated peptide receptor (FPR)-1, which is expressed, among other things, on neutrophils, and is closely associated with the activity of intestinal inflammation in UC and CD, as well as resistance to therapy with infliximab, ustekinumab, and vedolizumab [95]. Targeting pathways associated with neutrophil chemotaxis, such as CXCL8/CXCR1-2 and CD11b/CD18, etc., is also promising [96,97,98,99]. A separate rapidly developing direction for treating several immune-mediated diseases, including IBD and ASCVD, is targeting the formation of NETs. Among the agents that limit the formation of NETs, various PAD inhibitors, including PAD4 (GSK199, GSK484), and therapeutic antibodies that inhibit histone citrullination (anticitrullinated protein antibody), are at different stages of research [100]. Another direction in terms of a therapeutic effect on NETs is their dissolution, for example, using DNase I (or recombinant DNase I, Dornase alfa) [100,101]. In addition, TLR inhibitors, calcineurin inhibitors, ROS scavengers, and other agents are involved in various phases of clinical trials [102].

A potentially breakthrough approach to neutrophil targeting is neutrophil reprogramming, which enables the modulation of the phenotypic and functional characteristics of neutrophils without significantly compromising their protective functions. Thus, the use of 4-phenylbutyric acid (4-PBA), which restores peroxisome homeostasis, enables an increase in the number of resolving neutrophils (a shift in the subpopulation of neutrophils towards resolving neutrophils) expressing CD200R, CD86, and resolvin D1, which, in turn, is associated with a decrease in the volume of atherosclerotic lesions [103,104]. In the context of modulating neutrophil functions in IBD, influencing them through manipulations to the composition of the intestinal microbiome and microbiota-derived metabolites appears promising, which is described in detail in the review by C. Danne et al. [50].

Third, neutrophils represent a platform for targeted drug delivery in the development of site-specific therapies for IBD and atherosclerosis. For example, W. Li et al. developed a neutrophil membrane hybrid liposome nano-mimetic system (Ptdser-NM-Lipo/Fer-1) that effectively delivers Ferrostatin-1 (Fer-1) to atherosclerotic plaques and is composed of a Fer-1-loaded Ptdser-modified liposome core and a neutrophil shell [105]. Y. Liu et al. proposed a neutrophil membrane-coated zeolitic imidazolate framework-8 (ZIF-8) nanodelivery platform (AM@ZIF@NM) for the targeted transport of ASOs against microRNA-155 to endothelial cells in atherosclerotic lesions [106]. The use of neutrophil-based delivery systems in IBD is limited. Y.Z. Zhao et al. successfully used neutrophil membrane vesicles as a means of delivering the keratinocyte growth factor encapsulated in liposomes for treating UC [107].

## 6. Conclusions

Atherosclerosis in patients with chronic inflammatory diseases, including IBD, is the result of complex interactions among specific patterns involving immune response dysregulation, inflammation, and traditional cardiovascular risk factors [108]. The extrapolation of approaches for assessing and monitoring cardiovascular risk used in the general population to patients in this category is associated with a systematic underestimation of the relevant risks and the late initiation of preventive measures [109]. Progress in studying the role of neutrophils in the development of atherosclerosis and other cardiovascular complications in patients with IBD will contribute to the introduction of new biomarkers into clinical practice that provide the possibility of personalized assessment and monitoring of cardiovascular risk, the discovery of new therapeutic targets, and the planning of cross-disease, cross-discipline basket trials [110].

## Data Availability

Not applicable.

## References

[1] Zhou J.L., Bao J.C., Liao X.Y., Chen Y.J., Wang L.W., Fan Y.Y., Xu Q.Y., Hao L.X., Li K.J., Liang M.X. (2023). Trends and projections of inflammatory bowel disease at the global, regional and national levels, 1990–2050: A Bayesian age-period-cohort modeling study. BMC Public Health.

[2] Forbes A.J., Frampton C.M.A., Day A.S., Kaplan G.G., Gearry R.B. (2023). The epidemiology of inflammatory bowel disease in Oceania: A systematic review and meta-analysis of incidence and prevalence. Inflamm. Bowel. Dis..

[3] Wang S., Dong Z., Wan X. (2023). Global, regional, and national burden of inflammatory bowel disease and its associated anemia, 1990 to 2019 and predictions to 2050: An analysis of the global burden of disease study 2019. Autoimmun. Rev..

[4] Zhang Z.M., Lin Z.L., He B.X., Yan W.T., Zhang X.Y., Zhang Z.H., Wang L., Wang J.Q., Liu D.M., Zhang W. (2023). Epidemiological analysis reveals a surge in inflammatory bowel disease among children and adolescents: A global, regional, and national perspective from 1990 to 2019–insights from the China study. J. Glob. Health.

[5] Green Z., Ashton J.J., Rodrigues A., Spray C., Howarth L., Mallikarjuna A., Chanchlani N., Hart J., Bakewell C., Lee K.Y. (2024). Sustained increase in pediatric inflammatory bowel disease incidence across the South West United Kingdom over the last 10 years. Inflamm. Bowel Dis..

[6] Cao L., Dayimu A., Guan X., Duan M., Zeng S., Wang H., Zong J., Sun C., Yang X., Yang X. (2024). Global evolving patterns and cross-country inequalities of inflammatory bowel disease burden from 1990 to 2019: A worldwide report. Inflamm. Res..

[7] Kichloo A., El-Amir Z., Dahiya D.S., Wani F., Shaka H. (2021). Trends in hospitalizations and mortality for inflammatory bowel disease from a nationwide database study between 2008 and 2018. Bayl. Univ. Med Cent. Proc..

[8] Chen X., Xiang X., Xia W., Li X., Wang S., Ye S., Tian L., Zhao L., Ai F., Shen Z. (2023). Evolving trends and burden of inflammatory bowel disease in Asia, 1990–2019: A comprehensive analysis based on the Global Burden of Disease Study. J. Epidemiol. Glob. Health.

[9] Dupont-Lucas C., Leroyer A., Ley D., Spyckerelle C., Bertrand V., Turck D., Savoye G., Maunoury V., Guillon N., Fumery M. (2023). Increased risk of cancer and mortality in a large French population-based paediatric-onset inflammatory bowel disease retrospective cohort. J. Crohns Colitis.

[10] Follin-Arbelet B., Cvancarova Småstuen M., Hovde Ø., Jelsness-Jørgensen L.P., Moum B. (2023). Mortality in patients with inflammatory bowel disease: Results from 30 years of follow-up in a Norwegian inception cohort (the IBSEN study). J. Crohns Colitis.

[11] Shah N.N., Wass S., Hajjari J., Heisler A.C., Malakooti S., Janus S.E., Al-Kindi S.G. (2022). Proportionate cardiovascular mortality in chronic inflammatory disease in adults in the United States from 1999 to 2019. J. Clin. Rheumatol..

[12] Jaiswal V., Batra N., Dagar M., Butey S., Huang H., Chia J.E., Naz S., Endurance E.O., Raj N., Patel S. (2023). Inflammatory bowel disease and associated cardiovascular disease outcomes: A systematic review. Medicine.

[13] Groenendyk J.W., Rivera A.S., Sinha A., Lloyd-Jones D.M., Feinstein M.J. (2021). Changes in proportionate cardiovascular mortality in patients with chronic infectious and inflammatory conditions in the United States, 1999–2018. Sci. Rep..

[14] Sleutjes J.A.M., van Lennep J.E.R., van der Woude C.J., de Vries A.C. (2021). Thromboembolic and atherosclerotic cardiovascular events in inflammatory bowel disease: Epidemiology, pathogenesis, and clinical management. Ther. Adv. Gastroenterol..

[15] Sun H.H., Tian F. (2018). Inflammatory bowel disease and cardiovascular disease incidence and mortality: A meta-analysis. Eur. J. Prev. Cardiol..

[16] Nasir K., Acquah I., Dey A.K., Agrawal T., Hassan S.Z., Glassner K., Abraham B., Quigley E.M.M., Blankstein R., Virani S.S. (2022). Inflammatory bowel disease and atherosclerotic cardiovascular disease in U.S. adults—A population-level analysis in the National Health Interview Survey. Am. J. Prev. Cardiol..

[17] Lee M.T., Mahtta D., Chen L., Hussain A., Al Rifai M., Sinh P., Khalid U., Nasir K., Ballantyne C.M., Petersen L.A. (2021). Premature atherosclerotic cardiovascular disease risk among patients with inflammatory bowel disease. Am. J. Med..

[18] Alayo Q.A., Loftus E.V., Yarur A., Alvarado D., Ciorba M.A., de Las Fuentes L., Deepak P. (2023). Inflammatory bowel disease is associated with an increased risk of incident acute arterial events: Analysis of the United Kingdom Biobank. Clin. Gastroenterol. Hepatol..

[19] Fang L., Gao H., Gao X., Wu W., Miao Y., Zhang H., Guleng B., Zhang H., Wang Y., Li M. (2022). Risks of cardiovascular events in patients with inflammatory bowel disease in China: A retrospective multicenter cohort study. Inflamm. Bowel Dis..

[20] Livzan M.A., Bikbavova G.R., Lisyutenko N.S., Romanyuk A.E., Drapkina O.M. (2024). Cardiovascular Risk in Patients with Inflammatory Bowel Diseases-The Role of Endothelial Dysfunction. Diagnostics.

[21] Sleutjes J.A.M., Roeters van Lennep J.E., Verploegh P.J.P., van Doorn M.B.A., Vis M., Kavousi M., van der Woude C.J., de Vries A.C. (2022). Prevalence of ideal cardiovascular health and its correlates in patients with inflammatory bowel disease, psoriasis and spondyloarthropathy. Eur. J. Prev. Cardiol..

[22] Agrawal T., Acquah I., Dey A.K., Glassner K., Abraham B., Blankstein R., Virani S.S., Blaha M.J., Valero-Elizondo J., Mehta N. (2021). Prevalence of cardiovascular risk factors in a nationally representative adult population with inflammatory bowel disease without atherosclerotic cardiovascular disease. Am. J. Prev. Cardiol..

[23] Gravina A.G., Pellegrino R., Palladino G., Zanini A., Federico A., Zingone F. (2024). Too Many Couch Potatoes Among Middle-Aged Inflammatory Bowel Disease Patients: Findings from the “BE-FIT-IBD-2” Study. Gastroenterol. Insights.

[24] Gravina A.G., Pellegrino R., Durante T., Palladino G., D’Onofrio R., Mammone S., Arboretto G., Auletta S., Imperio G., Ventura A. (2023). Inflammatory bowel diseases patients suffer from significant low levels and barriers to physical activity: The “BE-FIT-IBD” study. World J. Gastroenterol..

[25] Kaazan P., Seow W., Yong S., Heilbronn L.K., Segal J.P. (2023). The Impact of Obesity on Inflammatory Bowel Disease. Biomedicines.

[26] Khakoo N.S., Ioannou S., Khakoo N.S., Vedantam S., Pearlman M. (2022). Impact of Obesity on Inflammatory Bowel Disease. Curr. Gastroenterol. Rep..

[27] Gravina A.G., Panarese I., Trotta M.C., D’Amico M., Pellegrino R., Ferraraccio F., Galdiero M., Alfano R., Grieco P., Federico A. (2024). Melanocortin 3,5 receptors immunohistochemical expression in colonic mucosa of inflammatory bowel disease patients: A matter of disease activity?. World J. Gastroenterol..

[28] Patel T.P., Jun J.Y., Seo A.Y., Levi N.J., Elizondo D.M., Chen J., Wong A.M., Tugarinov N., Altman E.K., Gehle D.B. (2025). Melanocortin 3 receptor regulates hepatic autophagy and systemic adiposity. Nat. Commun..

[29] Nuutinen S., Ailanen L., Savontaus E., Rinne P. (2018). Melanocortin overexpression limits diet-induced inflammation and atherosclerosis in LDLR^-/-^ mice. J. Endocrinol..

[30] Marín-Jiménez I., Carpio D., Hernández V., Muñoz F., Zatarain-Nicolás E., Zabana Y., Mañosa M., Rodríguez-Moranta F., Barreiro-de Acosta M., Gutiérrez Casbas A. (2025). Spanish Working Group in Crohn’s Disease and Ulcerative Colitis (GETECCU) position paper on cardiovascular disease in patients with inflammatory bowel disease. Gastroenterol. Hepatol..

[31] Hernández-Camba A., Carrillo-Palau M., Ramos L., Hernández Alvarez-Buylla N., Alonso-Abreu I., Hernández-Pérez A., Vela M., Arranz L., Hernández-Guerra M., González-Gay M.Á. (2021). Carotid plaque assessment reclassifies patients with inflammatory bowel disease into very-high cardiovascular risk. J. Clin. Med..

[32] Naami R., Tashtish N., Neeland I.J., Katz J., Sinh P., Nasir K., Chittajallu V., Mansoor E., Rajagopalan S., Al-Kindi S. (2023). Coronary artery calcium scoring for cardiovascular risk assessment in patients with inflammatory bowel disease. Am. Heart J..

[33] Mantaka A., Galanakis N., Tsetis D., Koutroubakis I.E. (2022). Abdominal aortic calcification in patients with inflammatory bowel disease: Does anti-tumor necrosis factor α use protect from chronic inflammation-induced atherosclerosis?. Intestig. Res..

[34] Sleutjes J.A.M., van der Woude C.J., Verploegh P.J.P., Aribas E., Kavousi M., Roeters van Lennep J.E., de Vries A.C. (2023). Cardiovascular risk profiles in patients with inflammatory bowel disease differ from matched controls from the general population. Eur. J. Prev. Cardiol..

[35] Alayo Q.A., Famutimi D., Ayoub M., De Las Fuentes L., Deepak P. (2024). Performance of ASCVD risk prediction models in individuals with inflammatory bowel disease: A UK Biobank study. Inflamm. Bowel Dis..

[36] Silvestre-Roig C., Braster Q., Ortega-Gomez A., Soehnlein O. (2020). Neutrophils as regulators of cardiovascular inflammation. Nat. Rev. Cardiol..

[37] Aroca-Crevillén A., Vicanolo T., Ovadia S., Hidalgo A. (2024). Neutrophils in physiology and pathology. Annu. Rev. Pathol..

[38] Garratt L.W. (2021). Current understanding of the neutrophil transcriptome in health and disease. Cells.

[39] Grieshaber-Bouyer R., Radtke F.A., Cunin P., Stifano G., Levescot A., Vijaykumar B., Nelson-Maney N., Blaustein R.B., Monach P.A., Nigrovic P.A. (2021). The neutrotime transcriptional signature defines a single continuum of neutrophils across biological compartments. Nat. Commun..

[40] Xie X., Shi Q., Wu P., Zhang X., Kambara H., Su J., Yu H., Park S.-Y., Guo R., Ren Q. (2020). Single-cell transcriptome profiling reveals neutrophil heterogeneity in homeostasis and infection. Nat. Immunol..

[41] Shah A.D., Denaxas S., Nicholas O., Hingorani A.D., Hemingway H. (2017). Neutrophil counts and initial presentation of 12 cardiovascular diseases: A CALIBER cohort study. J. Am. Coll. Cardiol..

[42] Welsh C., Welsh P., Mark P.B., Celis-Morales C.A., Lewsey J., Gray S.R., Lyall D.M., Iliodromiti S., Gill J.M.R., Pell J. (2018). Association of total and differential leukocyte counts with cardiovascular disease and mortality in the UK Biobank. Arter. Thromb. Vasc. Biol..

[43] Luo J., Thomassen J.Q., Nordestgaard B.G., Tybjærg-Hansen A., Frikke-Schmidt R. (2023). Neutrophil counts and cardiovascular disease. Eur. Heart J..

[44] Soehnlein O., Döring Y. (2023). Beyond association: High neutrophil counts are a causal risk factor for atherosclerotic cardiovascular disease. Eur. Heart J..

[45] Zhang X., Kang Z., Yin D., Gao J. (2023). Role of neutrophils in different stages of atherosclerosis. Innate Immun..

[46] Sreejit G., Johnson J., Jaggers R.M., Dahdah A., Murphy A.J., Hanssen N.M.J., Nagareddy P.R. (2022). Neutrophils in cardiovascular disease: Warmongers, peacemakers, or both?. Cardiovasc. Res..

[47] Herrero-Cervera A., Soehnlein O., Kenne E. (2022). Neutrophils in chronic inflammatory diseases. Cell Mol. Immunol..

[48] Tamassia N., Bianchetto-Aguilera F., Arruda-Silva F., Gardiman E., Gasperini S., Calzetti F., Cassatella M.A. (2018). Cytokine production by human neutrophils: Revisiting the “dark side of the moon”. Eur. J. Clin. Investig..

[49] Hall C.H.T., Campbell E.L., Colgan S.P. (2017). Neutrophils as components of mucosal homeostasis. Cell Mol. Gastroenterol. Hepatol..

[50] Danne C., Skerniskyte J., Marteyn B., Sokol H. (2024). Neutrophils: From IBD to the gut microbiota. Nat. Rev. Gastroenterol. Hepatol..

[51] Mortha A., Remark R., Del Valle D.M., Chuang L.S., Chai Z., Alves I., Azevedo C., Gaifem J., Martin J., Petralia F. (2022). Neutralizing anti-granulocyte macrophage-colony stimulating factor autoantibodies recognize post-translational glycosylations on granulocyte macrophage-colony stimulating factor years before diagnosis and predict complicated Crohn’s disease. Gastroenterology.

[52] Saez A., Herrero-Fernandez B., Gomez-Bris R., Sanchez-Martinez H., Gonzalez-Granado J.M. (2023). Pathophysiology of inflammatory bowel disease: Innate immune system. Int. J. Mol. Sci..

[53] Segal A.W. (2018). The role of neutrophils in the pathogenesis of Crohn’s disease. Eur. J. Clin. Investig..

[54] Denson L.A., Jurickova I., Karns R., Shaw K.A., Cutler D.J., Okou D., Valencia C.A., Dodd A., Mondal K., Aronow B.J. (2019). Genetic and transcriptomic variation linked to neutrophil granulocyte-macrophage colony-stimulating factor signaling in pediatric Crohn’s disease. Inflamm. Bowel Dis..

[55] Bamias G., Zampeli E., Domènech E. (2022). Targeting neutrophils in inflammatory bowel disease: Revisiting the role of adsorptive granulocyte and monocyte apheresis. Expert. Rev. Gastroenterol. Hepatol..

[56] Parigi T.L., Cannatelli R., Nardone O.M., Zammarchi I., Shivaji U., Furfaro F., Zardo D., Spaggiari P., Del Sordo R., Setti O. (2023). Neutrophil-only histological assessment of ulcerative colitis correlates with endoscopic activity and predicts long-term outcomes in a multicentre study. J. Crohn’s Colitis.

[57] Pai R.K., Hartman D.J., Rivers C.R., Regueiro M., Schwartz M., Binion D.G., Pai R.K. (2020). Complete resolution of mucosal neutrophils associates with improved long-term clinical outcomes of patients with ulcerative colitis. Clin. Gastroenterol. Hepatol..

[58] Zhang C., Zhang J., Zhang Y., Song Z., Bian J., Yi H., Ma Z. (2023). Identifying neutrophil-associated subtypes in ulcerative colitis and confirming neutrophils promote colitis-associated colorectal cancer. Front. Immunol..

[59] Magalhães D., Peyrin-Biroulet L., Estevinho M.M., Danese S., Magro F. (2023). Pursuing neutrophils: Systematic scoping review on blood-based biomarkers as predictors of treatment outcomes in inflammatory bowel disease. Ther. Adv. Gastroenterol..

[60] Juzenas S., Hübenthal M., Lindqvist C.M., Kruse R., Steiert T.A., Degenhardt F., Schulte D., Nikolaus S., Zeissig S., Bergemalm D. (2022). Detailed transcriptional landscape of peripheral blood points to increased neutrophil activation in treatment-naïve inflammatory bowel disease. J. Crohn’s Colitis.

[61] van Unen V., Ouboter L.F., Li N., Schreurs M., Abdelaal T., Kooy-Winkelaar Y., Beyrend G., Höllt T., Maljaars P.W.J., Mearin M.L. (2022). Identification of a disease-associated network of intestinal immune cells in treatment-naïve inflammatory bowel disease. Front. Immunol..

[62] Fu X., Liu H., Huang G., Dai S.S. (2021). The emerging role of neutrophils in autoimmune-associated disorders: Effector, predictor, and therapeutic targets. Med. Commun..

[63] Wigerblad G., Kaplan M.J. (2023). Neutrophil extracellular traps in systemic autoimmune and autoinflammatory diseases. Nat. Rev. Immunol..

[64] Bissenova S., Ellis D., Mathieu C., Gysemans C. (2022). Neutrophils in autoimmunity: When the hero becomes the villain. Clin. Exp. Immunol..

[65] Sadeghi M., Dehnavi S., Jamialahmadi T., Johnston T.P., Sahebkar A. (2023). Neutrophil extracellular trap: A key player in the pathogenesis of autoimmune diseases. Int. Immunopharmacol..

[66] Xu G., Zhang W., Yang J., Sun N., Qu X. (2023). Identification of neutrophil extracellular traps and crosstalk genes linking inflammatory bowel disease and osteoporosis by integrated bioinformatics analysis and machine learning. Sci. Rep..

[67] Ding Y., Yang Y., Xue L. (2023). Immune cells and their related genes provide a new perspective on the common pathogenesis of ankylosing spondylitis and inflammatory bowel diseases. Front. Immunol..

[68] Jatana S., Ponti A.K., Johnson E.E., Rebert N.A., Smith J.L., Fulmer C.G., Maytin E.V., Achkar J.P., Fernandez A.P., McDonald C. (2023). A novel murine model of pyoderma gangrenosum reveals that inflammatory skin-gut crosstalk is mediated by IL-1β-primed neutrophils. Front. Immunol..

[69] He R., Zhao S., Cui M., Chen Y., Ma J., Li J., Wang X. (2023). Cutaneous manifestations of inflammatory bowel disease: Basic characteristics, therapy, and potential pathophysiological associations. Front. Immunol..

[70] Kaneko R., Matsui A., Watanabe M., Harada Y., Kanamori M., Awata N., Kawazoe M., Takao T., Kobayashi Y., Kikutake C. (2023). Increased neutrophils in inflammatory bowel disease accelerate the accumulation of amyloid plaques in the mouse model of Alzheimer’s disease. Inflamm. Regen..

[71] Wang Q., Guo Q., Zhou L., Li W., Yuan Y., Lei W., Liu K., Xu M., Diao T., Gao H. (2022). Associations of baseline and changes in leukocyte counts with incident cardiovascular events: The Dongfeng-Tongji Cohort Study. J. Atheroscler. Thromb..

[72] Yang W., Wu S., Xu F., Shu R., Song H., Chen S., Shao Z., Cui L. (2023). Distinct WBC trajectories are associated with the risks of incident CVD and all-cause mortality. J. Atheroscler. Thromb..

[73] Feng W., Liu Y., Zhu L., Xu L., Shen H. (2022). Evaluation of neutrophil-to-lymphocyte ratio and platelet-to-lymphocyte ratio as potential markers for ulcerative colitis: A retrospective study. BMC Gastroenterol..

[74] Kurimoto N., Nishida Y., Hosomi S., Itani S., Kobayashi Y., Nakata R., Ōminami M., Nadatani Y., Fukunaga S., Ōtani K. (2023). Neutrophil-to-lymphocyte ratio may predict clinical relapse in ulcerative colitis patients with mucosal healing. PLoS ONE.

[75] Pang W., Zhang B., Jin L., Yao Y., Han Q., Zheng X. (2023). Serological biomarker-based machine learning models for predicting the relapse of ulcerative colitis. J. Inflamm. Res..

[76] Zhou G.X., Liu Z.J. (2017). Potential roles of neutrophils in regulating intestinal mucosal inflammation of inflammatory bowel disease. J. Dig. Dis..

[77] Luan Y., Hu J., Wang Q., Wang X., Li W., Qu R., Yang C., Rajendran B.K., Zhou H., Liu P. (2024). Wnt5 controls splenic myelopoiesis and neutrophil functional ambivalency during DSS-induced colitis. Cell Rep..

[78] Bénard A., Mittelstädt A., Klösch B., Glanz K., Müller J., Schoen J., Nüse B., Brunner M., Naschberger E., Stürzl M. (2023). IL-3 orchestrates ulcerative colitis pathogenesis by controlling the development and the recruitment of splenic reservoir neutrophils. Cell Rep..

[79] Arosa L., Camba-Gómez M., Conde-Aranda J. (2022). Neutrophils in intestinal inflammation: What we know and what we could expect for the near future. Gastrointest. Disord..

[80] Libby P., Hansson G.K. (2019). From focal lipid storage to systemic inflammation: JACC Review Topic of the Week. J. Am. Coll. Cardiol..

[81] Ostendorf Y., Rolauer L., Pasch N., Schaefer H., Heitmann S., Petzsch P., Poschmann G., Hartwig S., Lehr S., Koehrer K. (2022). Neutrophils as major drivers of increased atherosclerosis in a murine model of chronic colitis. Eur. Heart J..

[82] Yao Z., Zhang B., Niu G., Yan Z., Tong X., Zou Y., Li Y., Yang M. (2022). Neutrophil infiltration characterized by upregulation of *S100A8, S100A9, S100A12*, and CXCR2 is associated with the co-occurrence of Crohn’s disease and peripheral artery disease. Front. Immunol..

[83] Tang X., Zhou Y., Chen Z., Liu C., Wu Z., Zhou Y., Zhang F., Lu X., Tang L. (2024). Identification of key biomarkers for predicting CAD progression in inflammatory bowel disease via machine-learning and bioinformatics strategies. J. Cell. Mol. Med..

[84] Luo X., Wang R., Zhang X., Wen X., Deng S., Xie W. (2023). Identification of *CCL2, CXCR2, S100A9* as immune-related gene markers and immune infiltration characteristics of inflammatory bowel disease and heart failure via bioinformatics analysis and machine learning. Front. Cardiovasc. Med..

[85] Fu Q., Shen T., Qiu W., Liao Y., Yu M., Zhou Y. (2025). FOSB is a key factor in the genetic link between inflammatory bowel disease and acute myocardial infarction: Multiple bioinformatics analyses and validation. BMC Med. Genom..

[86] Wu M., Liu D., Xiong X., Su Q., Xiang Y., Shen L., An Z., Yang X. (2025). Analysis of the molecular mechanisms of ulcerative colitis and atherosclerosis by microarray data. Sci. Rep..

[87] Buso G., Faggin E., Bressan A., Galliazzo S., Cinetto F., Felice C., Fusaro M., Erdmann A., Pauletto P., Rattazzi M. (2023). Biomarkers of neutrophil activation in patients with symptomatic chronic peripheral artery disease predict worse cardiovascular outcome. Biomedicines.

[88] Swaminathan A., Borichevsky G.M., Frampton C.M., Day A.S., Hampton M.B., Kettle A.J., Gearry R.B. (2024). Comparison of fecal calprotectin and myeloperoxidase in predicting outcomes in inflammatory bowel disease. Inflamm. Bowel Dis..

[89] Ling Lundström M., Peterson C., Lampinen M., Hedin C.R.H., Keita Å.V., Kruse R., Magnusson M.K., Lindqvist C.M., Repsilber D., D’Amato M. (2023). Fecal biomarkers of neutrophil and eosinophil origin reflect the response to biological therapy and corticosteroids in patients with inflammatory bowel disease. Clin. Transl. Gastroenterol..

[90] Pavlidis P., Tsakmaki A., Pantazi E., Li K., Cozzetto D., Bell J.D., Yang F., Lo J.W., Alberts E., Sa A.C.C. (2022). Interleukin-22 regulates neutrophil recruitment in ulcerative colitis and is associated with resistance to ustekinumab therapy. Nat. Commun..

[91] Zhou Z., Zhang Y., Yang X., Pan Y., Li L., Gao C., He C. (2022). Clinical significance of novel neutrophil-based biomarkers in the diagnosis and prediction of response to infliximab therapy in Crohn’s disease. Front. Immunol..

[92] Friedrich M., Pohin M., Jackson M.A., Korsunsky I., Bullers S.J., Rue-Albrecht K., Christoforidou Z., Sathananthan D., Thomas T., Ravindran R. (2021). IL-1-driven stromal-neutrophil interactions define a subset of patients with inflammatory bowel disease that does not respond to therapies. Nat. Med..

[93] Everett B.M., MacFadyen J.G., Thuren T., Libby P., Glynn R.J., Ridker P.M. (2020). Inhibition of interleukin-1β and reduction in atherothrombotic cardiovascular events in the CANTOS trial. J. Am. Coll. Cardiol..

[94] Aggeletopoulou I., Kalafateli M., Tsounis E.P., Triantos C. (2024). Exploring the role of IL-1β in inflammatory bowel disease pathogenesis. Front. Med..

[95] McAllister M.J., Hall R., Whelan R.J., Fischer L.J., Chuah C.S., Cartlidge P.D., Drury B., Rutherford D.G., Duffin R.M., Cartwright J.A. (2024). Formylated peptide receptor-1-mediated gut inflammation as a therapeutic target in inflammatory bowel disease. Crohns Colitis 360.

[96] Dhayni K., Zibara K., Issa H., Kamel S., Bennis Y. (2022). Targeting CXCR1 and CXCR2 receptors in cardiovascular diseases. Pharmacol. Ther..

[97] Xie Y., Kuang W., Wang D., Yuan K., Yang P. (2023). Expanding role of CXCR2 and therapeutic potential of CXCR2 antagonists in inflammatory diseases and cancers. Eur. J. Med. Chem..

[98] Kelm M., Lehoux S., Azcutia V., Cummings R.D., Nusrat A., Parkos C.A., Brazil J.C. (2020). Regulation of neutrophil function by selective targeting of glycan epitopes expressed on the integrin CD11b/CD18. FASEB J..

[99] Zhou G., Zhu F., Zhang H., Wang Y., Yang Y., Jin G., Wang Y., Dong G., Xiong H. (2023). PTK2B regulates immune responses of neutrophils and protects mucosal inflammation in ulcerative colitis. FASEB J..

[100] Gu C., Pang B., Sun S., An C., Wu M., Wang N., Yuan Y., Liu G. (2023). Neutrophil extracellular traps contributing to atherosclerosis: From pathophysiology to clinical implications. Exp. Biol. Med..

[101] Wang C.J., Ko G.R., Lee Y.Y., Park J., Park W., Park T.E., Jin Y., Kim S.N., Lee J.S., Park C.G. (2024). Polymeric DNase-I nanozymes targeting neutrophil extracellular traps for the treatment of bowel inflammation. Nano Converg..

[102] Huang J., Hong W., Wan M., Zheng L. (2022). Molecular mechanisms and therapeutic target of NETosis in diseases. Med. Commun..

[103] Geng S., Zhang Y., Lee C., Li L. (2019). Novel reprogramming of neutrophils modulates inflammation resolution during atherosclerosis. Sci. Adv..

[104] Lin R., Yi Z., Wang J., Geng S., Li L. (2022). Generation of resolving memory neutrophils through pharmacological training with 4-PBA or genetic deletion of *TRAM*. Cell Death Dis..

[105] Li W., Liu C., Wang S., Liu N. (2023). Neutrophil membrane biomimetic delivery system (Ptdser-NM-Lipo/Fer-1) designed for targeting atherosclerosis therapy. IET Nanobiotechnology.

[106] Liu Y., He M., Yuan Y., Nie C., Wei K., Zhang T., Chen T., Chu X. (2023). Neutrophil-membrane-coated biomineralized metal-organic framework nanoparticles for atherosclerosis treatment by targeting gene silencing. ACS Nano.

[107] Zhao Y.Z., ZhuGe D.L., Tong M.Q., Lin M.T., Zheng Y.W., Jiang X., Yang W.G., Yao Q., Xiang Q., Li X.K. (2019). Ulcerative colitis-specific delivery of keratinocyte growth factor by neutrophils-simulated liposomes facilitates the morphologic and functional recovery of the damaged colon through alleviating the inflammation. J. Control. Release.

[108] Yennemadi A.S., Jordan N., Diong S., Keane J., Leisching G. (2024). The link between dysregulated immunometabolism and vascular damage: Implications for the development of atherosclerosis in systemic lupus erythematosus and other rheumatic diseases. J. Rheumatol..

[109] Legge A., Hanly J.G. (2018). Managing premature atherosclerosis in patients with chronic inflammatory diseases. Can. Med. Assoc. J..

[110] Hosack T., Thomas T., Ravindran R., Uhlig H.H., Travis S.P.L., Buckley C.D. (2023). Inflammation across tissues: Can shared cell biology help design smarter trials?. Nat. Rev. Rheumatol..

